# Fibronectin on circulating extracellular vesicles as a liquid biopsy to detect breast cancer

**DOI:** 10.18632/oncotarget.9561

**Published:** 2016-05-23

**Authors:** Pyong-Gon Moon, Jeong-Eun Lee, Young-Eun Cho, Soo Jung Lee, Yee Soo Chae, Jin Hyang Jung, In-San Kim, Ho Yong Park, Moon-Chang Baek

**Affiliations:** ^1^ Department of Molecular Medicine, Cell and Matrix Research Institute, School of Medicine, Kyungpook National University, Daegu 700-422, Republic of Korea; ^2^ Department of Oncology/Hematology, Kyungpook National University Hospital, Daegu 700-721, Republic of Korea; ^3^ Department of Breast & Thyroid Surgery, Kyungpook National University Hospital, Daegu 700-721, Republic of Korea; ^4^ Center for Theragnosis, Biomedical Research Institute, Korea Institute of Science and Technology, KU-KIST School, Korea University, Seoul 02841, Republic of Korea

**Keywords:** breast cancer, diagnosis, extracellular vesicle, ELISA

## Abstract

Extracellular vesicles (EVs) secreted from cancer cells have potential for generating cancer biomarker signatures. Fibronectin (FN) was selected as a biomarker candidate, due to the presence in surface on EVs secreted from human breast cancer cell lines. A subsequent study used two types of enzyme-linked immunosorbent assays (ELISA) to determine the presence of these proteins in plasma samples from disease-free individuals (n=70), patients with BC (n=240), BC patients after surgical resection (n=40), patients with benign breast tumor (n=55), and patients with non-cancerous diseases (thyroiditis, gastritis, hepatitis B, and rheumatoid arthritis; n=80). FN levels were significantly elevated (p<. 0001) at all stages of BC, and returned to normal after tumor removal. The diagnostic accuracy for FN detection in extracellular vesicles (ELISA method 1) (area under the curve, 0.81; 95% CI, 0.76 to 0.86; sensitivity of 65.1% and specificity of 83.2%) were also better than those for FN detection in the plasma (ELISA method 2) (area under the curve, 0.77; 95% CI, 0.72 to 0.83; sensitivity of 69.2% and specificity of 73.3%) in BC. The diagnostic accuracy of plasma FN was similar in both the early-stage BC and all BC patients, as well as in the two sets. This liquid biopsy to detect FN on circulating EVs could be a promising method to detect early breast cancer.

## INTRODUCTION

Breast cancer (BC) is the leading cause of death worldwide, causing up to half-a-million deaths yearly, and is the most common type of cancer in women [1–3]. It is valuable to identify an early biomarker to detect breast cancer because patients can live longer with less extensive treatment when the cancer is detected early [4]. This cancer metastasizes when cancer cells break through the duct or glandular walls to invade the surrounding tissues of the breast and enter the bloodstream, where they can travel to distant organs. Metastatic BC, classified as stage IV disease, is usually diagnosed when BC has recurred, months or years after treatment for earlier-stage disease. Invasion and metastasis are continuing therapeutic challenges and common causes of death for patients with cancer [5]. Although it is clear that they have complex processes, including mesenchymal movement, amoeboid locomotion, and migration through tissues, the molecular mechanisms are still poorly understood [6, 7]. However, outside-in signaling triggered by binding of integrin [8] with extracellular matrix proteins such as fibronectin (FN) [9] plays an essential role in this process. FN binds multiple integrins [10], resulting in the activation of various signaling proteins, including focal adhesion kinase (FAK) [11], Src [12], and Akt [13]. Cells activated by these signals express matrix metalloproteinases [14], become migratory [15], and invade basement membranes [16]. An improved understanding of the molecular basis underlying cancer invasion and metastasis is essential to develop effective targets for therapy.

Extracellular vesicles (EVs) are membranous vesicles that are secreted by various cells, and have been classified into several sub-categories, including exosomes (50-100 nm in diameter) and microvesicles (100-1,000 nm in diameter) [17, 18]. Cancer cells, and neighboring cells in the tumor microenvironment, secrete EVs that play important roles in pro-metastatic signaling, angiogenesis, and immune suppression in an autocrine or paracrine manner [19–22]. It has been also suggested that cancer cell-derived EVs have the potential to be used as early biomarkers for various cancers because the membrane vesicles are secreted continuously from the early stage of disease into body fluids, including blood [23–25].

Based on this background information, we hypothesized that proteins in EVs can be used as early diagnostic biomarkers for BC. We employed a proteomic approach to identify FN as disease-specific proteins on EVs isolated from two human BC cell lines. We then confirmed that the levels of FN, measured by two different ELISAs in plasma, correlated with the presence of BC.

## RESULTS

### Identification of FN on EVs from breast cancer cells

**Figure 1:**
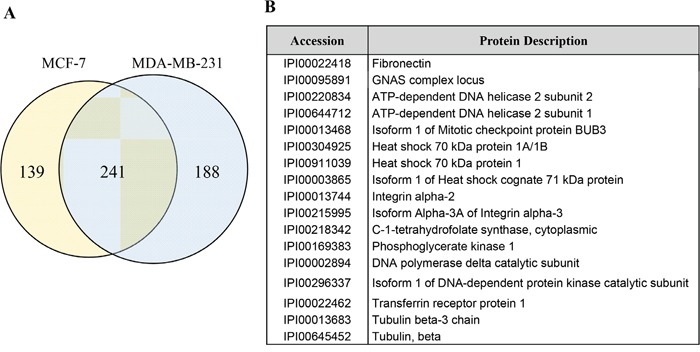
Identification of fibrinectin on extracellular vesicles (EVs) from breast cancer cells **A.** EVs from breast cancer cells, were analyzed using LC-MS/MS for identification of biomarker candidates. Numbers of proteins identified EV from MCF-7 and MDA-MB-231 were shown as Venn diagrams. **B.** In 241 common proteins, 17 proteins related to cancer development were selected.

To identify biomarker candidates for BC, EVs isolated from two representative human BC cell lines were analyzed using proteomics [26, 27]. Among 568 proteins (Supplementary Table 1 and 2), 241 proteins were identified both EVs from MDA-MB-231 and MCF-7 (Supplementary Table 3, Figure 1A and 1B). We searched all 241 proteins in the DAVID bioinformatics resource database and selected 17 proteins which are related to cancer development. In addition, 4 proteins (FN, GNAS complex locus, Heat shock cognate 71 kDa protein, and Transferrin receptor protein 1) are located in EVs. Finally, FN was selected because they can be easily detected using appropriate antibodies due to outer-vesicle location [21].

### Characteristics of participants

**Figure 2:**
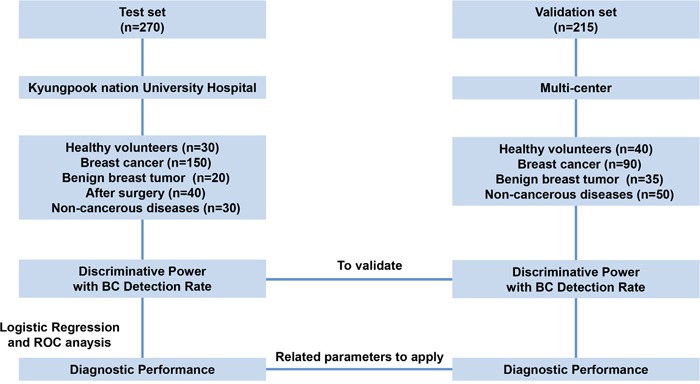
Overview of the study design Fibronectin was evaluated as a novel biomarker for the early detection of BC using plasma (1 μL) from test cohort (*n* = 270) and validation cohort (*n* = 215).

A total of 240 BC patients and 205 controls were eligible for this study. The mean age for the participants was 51 years. Demographic characteristics were well balanced between the two groups (Table 1). This study included 37, 58, 81, 54, and 8 BC patients with stage 0, I, II, III, and IV, respectively. Among them, 176 (74.3%) of the BC patients were in early-stage (0, I, and II) of the disease. There were 205 non-cancer individuals, including 70 healthy subjects, 55 benign breast tumors (bB) patients, and 80 non-cancerous diseases (NC) patients. Additionally, samples from 40 BC patients that underwent surgery were used (Figure 2).

**Table 1:** Characteristics of study population

Characteristic	Test				Validation				Total		P^*^
	No.	%	Mean	SD	No.	%	Mean	SD	No.	%	
Number of Study Population	270	56.9			215	43.1			485		
Age, years			51.2	11.4			51.9	12.3			.8090^**^
Breast cancer	150	62.8			90	37.2			240		
Histological grade^†^											.0148^#^
1 2 3	24 89 37	17.9 56.8 25.4			16 36 38	19.0 40.0 41.0			40 125 75	18.2 50.6 31.2	
Stage^‡^											<.0001^#^
0 I II III IV	33 43 43 21 10	22.0 28.7 28.7 14.0 6.6			4 15 38 33	5.0 18.0 42.0 35.0			37 58 81 54 10	15.4 24.2 33.7 22.5 4.2	
Estrogen receptor (ER) ^¶^											.2045^#^
Negative Positive	33 117	24.9 75.1			27 63	32.0 68.0			60 180	27.5 72.5	
Progesterone receptor (PgR) ^¶^											.7049^#^
Negative Positive	47 103	33.7 66.3			31 59	36.0 64.0			78 162	34.6 65.4	
HER2^¶^											.4544^#^
Positive^§^ Negative	24 126	20.1 79.9			21 69	24.0 76.0			45 195	21.6 78.4	
Healthy control	30	43.2			40	56.8			70		
Non-cancerous diseases	30				50				80		.4635^#^
Thyroiditis Gastritis Hepatitis B Rheumatoid arthritis	5 5 4 16	20 17.5 15 47.5			7 5 5 33	13.8 12.1 10.3 63.8			12 12 9 49	16.3 14.3 12.2 57.1	
After surgery	40								40		
Benign breast tumor	20	40.6			35	59.4			55		

* *P* value comparing test and validation groups.

** *t*-test.

# *χ*^2^ test.

† modified Scarff-Bloom-Richardson grading system.

‡ 7th edition of AJCC staging system.

¶ according to immunohistochemical (IHC) staining of ER, PgR, and HER2.

§ IHC (3+) or FISH (+).

### Levels of FN determined by two different ELISAs in the plasma of BC patients

**Figure 3:**
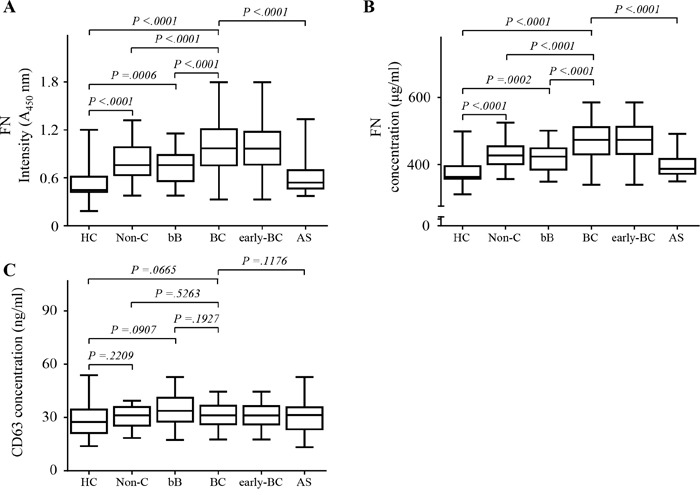
Fibronectin (FN) levels in plasma in the test set using two types of enzyme-linked immunosorbent assays (ELISA) in test set **A.** FN levels on circulating extracellular vesicles in plasma using Method 1. **B.** FN levels in plasma using Method 2. **C.** CD63 levels in plasma. All data were obtained using samples from the same subjects. Healthy controls (HC), *n* = 30; non-cancerous diseases (Non-C), *n* = 30; benign breast tumors (bB), *n* = 20; breast cancer (BC), *n* = 150; early-stage breast cancer (early-BC), *n* = 119; after surgery (AS), *n* = 40. The black horizontal lines are means, and error bars are standard errors.

FN levels in 1 μL of plasma from all patients were assessed by two different ELISAs using different primary capture antibodies (Method 1; monoclonal anti-CD63 antibody to capture circulating EVs and Method 2; polyclonal FN antibody to capture plasma FN). We recruited 485 participants overall: 270 in the test set and 215 in the validation set (Figure 2, Table 1). FN levels in plasma were significantly higher in patients with BC in the test set than in all controls (absorbance at 450 nm (A_450_) median 0.84, interquartile range (IQR) 0·75–1·57; mean 1.1, standard deviation (SD) 0·33; *p* <0·0001; Figure 3A) including healthy control (A_450_ median 0.45, IQR 0·42–0.62; mean 0.53, SD 0·22; *p* <0·0001; Figure 3A); values differ significantly between the disease control groups (benign breast tumors n=20, and non-cancerous diseases n=30) and health control (Figure 3A and 3B, (healthy controls n=30,). 40 plasma samples were collected from BC patients after surgical resection. The mean level of FN in plasma from patients with BC was 0.85 (SD 0·28), and values dropped after surgical resection (0·53 [0·21], *p* =0·00015; Figure 3). Although the levels of FN were increased in BC patients compared to control, the levels of CD63, a representative exosome marker protein, were unchanged in BC patients relative to control (Figure 3C).

The levels of FN neither correlate with the stage of the breast tumors (Supplementary Figure 1) nor correlate with the size of the breast tumors (Spearman *r* = 0.079; *p*= .306) (Supplementary Figure 2A). Moreover, FN levels showed no significant correlation with the status of four types of receptors in BCs (ER/PR+Her2+, ER/PR+Her2-, ER/PR-Her2+, ER/PR-Her2-; p > .05; Supplementary Figure 2B).

### Sensitivities and specificities of FN for BC diagnosis

The 150 BC patients were categorized according to AJCC stages (Table 1) and compared plasma levels of FN in each stage (Figure 3A and 3B). Our results showed that plasma levels of FN levels in BC patients with advanced stage III/IV were markedly elevated. Furthermore, there was the significant difference between plasma levels of FN levels in patients with early-stage BC (stages 0, I, and II) and those in healthy controls (*p* < 0.001).

**Figure 4:**
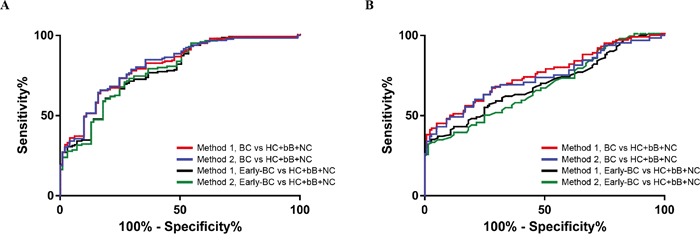
Diagnostic outcomes for fibrinectin (FN) in the diagnosis of breast cancer (BC) using ELISA method 1 and 2 **A.** ROC curves for FN for all patients with BC versus three control groups in test set. **B.** ROC curves for FN for all patients with BC versus three control groups in validation set.

The diagnostic value of plasma levels of FN was evaluated by ROC curves analysis, and sensitivity, specificity, and all cutoff values of FN levels were determined. Comparing BC patients with controls, the best cutoff level of FN was 0.738 (A_450_) and 529.54 ng/mL for each method. The cutoff of 0.738 (A_450_) and 529.54 ng/mL were selected to categorize patients as higher or lower plasma FN level for two ELISA methods. Results for FN were showed in the diagnosis of BC (Table 2, Figure 4). Comparing disease control group (benign breast tumors n=20, and non-cancerous diseases n=30) with healthy controls, the cutoff level of FN was 0.764 (A_450_). The cutoff of 0.613 (A_450_) were selected to categorize patients with BC compared to healthy control. The area under the curve (AUC) for exosomal FN using ELISA 1 (0.810, 95% CI: 0·758–0·862, sensitivity 65.1%, specificity of 83.2%) was greater than plasma FN using ELISA 2 (0.773, 95% confidence interval (CI): 0.721–0.834, sensitivity, 69.2%; specificity, 73.3%). After excluding HC, the AUC for exosomal FN using ELISA 1 (0.746, 95% CI 0.680-0.811) was also greater than plasma FN using ELISA 2 (0.710, 95% CI: 0.641-0.799). For early-stage BC, the AUC for exosomal FN using ELISA 1 was greater than that of plasma FN using ELISA 2, regardless of HC inclusion or exclusion. In addition, the sensitivity and specificity for exosomal FN using ELISA 1 were also better than those for plasma FN using ELISA 2 (Table 2).

**Table 2:** Results for measurement of plasma fibronectin using enzyme-linked immunosorbent assays (ELISA) in the diagnosis of breast cancer in test set

	AUC (95%CI)	Sensitivity (%)	Specificity (%)	LR +	NR −
Method 1					
BC *vs* HC+bB+NC^*^	0.810 (0.758-0.862)	65.1%	83.2%	3.88	0.42
BC *vs* bB+NC	0.746 (0.680-0.811)	63.4%	78.8%	2.99	0.46
Early-BC *vs* HC+bB+NC	0.815 (0.761-0.869)	64.4%	84.2%	4.08	0.42
Early−BC *vs* bB+NC	0.754 (0.685-0.822)	65.9%	77.3%	2.90	0.44
Method 2					
BC *vs* HC+bB+NC	0.773 (0.721-0.834)	69.2%	73.3%	2.59	0.42
BC *vs* bB+NC	0.710 (0.641-0.779)	60.4%	75.8%	2.49	0.52
Early-BC *vs* HC+bB+NC	0.779 (0.719-0.838)	72.7%	71.3%	2.53	0.38
Early−BC *vs* bB+NC	0.713 (0.640-0.787)	59.9%	75.8%	2.47	0.53

* BC, breast cancer; HC, Healthy controls; bB, benign breast tumors; NC, non-cancerous diseases; Early−- BC, early stage breast cancer.

### Diagnostic performance of FN in validation set

**Figure 5:**
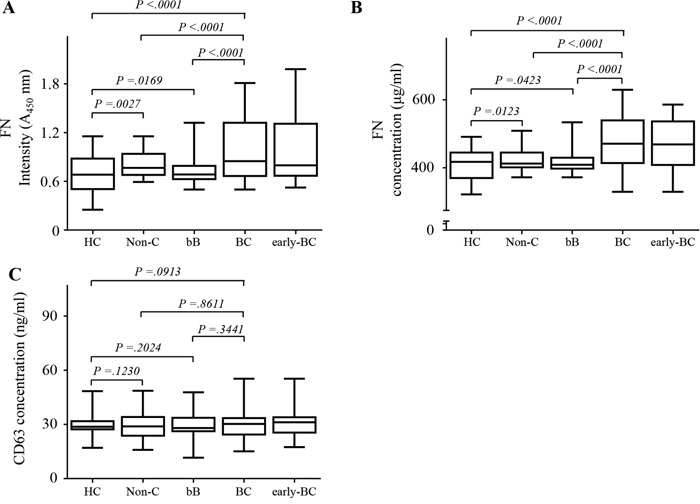
Fibronectin (FN) levels in plasma in the validation set using two types of enzyme-linked immunosorbent assays (ELISA) in validation set **A.** FN levels on circulating EVs in plasma using Method 1. **B.** FN levels in plasma using Method 2. **C.** CD63 levels in plasma. All data were obtained using samples from the same subjects. Healthy controls (HC), *n* = 40; non-cancerous diseases (Non-C), *n* = 50; benign breast tumors (bB), *n* = 35; breast cancer (BC), *n* = 90; early-stage breast cancer (early-BC), *n* = 57. The black horizontal lines are means, and error bars are SEs.

Using FN threshold values of 0.738 (A_450_) for ELISA Method 1 and 529.54 ng/mL for ELISA Method 2, we observed similar results in the validation set to those in the test set. A patients with BC in the validation set were positive for FN using ELISA method 1 than ELISA method 2 (62 [68.9%] vs. 49 [54.2%] of 90 patients; Figure 5A and 5B). The levels of CD63 were unchanged in BC patients relative to control (Figure 5C). In the assessment of differential diagnostic accuracy, exosomal FN using ELISA method 1 (AUC, 0.748; 95% CI 0.683–0.812; sensitivity, 68.9%; specificity, 72.0%) had slightly higher AUC values than ELISA method 2 (AUC, 0.684; 95% CI 0.614–0.753; sensitivity, 54.4%; specificity, 75.2%) in patients with BC, compared to patients with benign breast tumors and non-cancerous diseases (Figure 5B and Table 3).

**Table 3:** Results for measurement of plasma fibronectin using enzyme-linked immunosorbent assays (ELISA) in the diagnosis of breast cancer in validation set

	AUC (95%CI)	Sensitivity (%)	Specificity (%)	LR +	NR −
Method 1					
BC *vs* HC+bB+NC	0.748 (0.683-0.812)	68.9%	72.0%	2.43	0.44
BC *vs* bB+NC	0.736 (0.666-0.806)	66.1%	74.2%	2.56	0.46
Early-BC *vs* HC+bB+NC	0.737 (0.657-0.812)	67.7%	72.0%	2.42	0.45
Early−BC *vs* bB+NC	0.722 (0.637-0.807)	67.7%	74.3%	2.63	0.43
Method 2					
BC *vs* HC+bB+NC	0.684 (0.614-0.753)	54.4%	75.2%	2.19	0.61
BC *vs* bB+NC	0.665 (0.589-0.741)	56.0%	75.7%	2.30	0.58
Early-BC *vs* HC+bB+NC	0.672 (0.591-0.753)	49.2%	75.2%	1.98	0.68
Early−BC *vs* bB+NC	0.654 (0.566-0.743)	49.2%	76.7%	2.11	0.66

* BC, breast cancer; HC, Healthy controls; bB, benign breast tumors; NC, non-cancerous diseases; Early−BC, early stage breast cancer.

## DISCUSSION

EVs contain many disease-associated proteins, giving important information [28]. Expression profiling of EV, including miRNA and proteins associated with disease has been explored where the majority of researchers have used peripheral blood [29, 30] or cell-free serum or plasma [31, 32]. Recently, an increasing number of exosomal proteins have been found to be potential biomarkers for a variety of diseases, including cancer [33] as well as kidney diseases [25, 34]. These exosomal proteins may have great potential in clinical diagnostics and should be further explored. Chen *et al.* identified that 24 exosomal proteins were presented at significantly different levels between bladder cancer and control patients [35].

Although these sources are rich in miRNA and proteins, it can be difficult to differentiate disease-specific miRNA and proteins biomarkers from those expressed both in healthy and diseased patients. Comparing miRNA and proteins detection from whole serum and isolated exosomes showed that EV isolation improves the sensitivity of miRNA and proteins amplification from human biologic fluids. Therefore, in this study, the level of FN on EV using Method 1 (AUC, 0.810; 95% CI, 0.758–0.862 vs. 0.748, 0.683–0.812, *p=* 0.166; Figure 4, Table 2 and 3) yielded an improved differential diagnosis of BC from all controls compared with the level of FN in plasma using Method 2 (AUC, 0.773; 95% CI, 0.721–0.834 vs. 0.684, 0.614–0.753, *p=* 0.091; Figure 4, Table 2 and 3).

The groups differed to some degree in results for diagnostic performance (Table 2 and 3). For instance, the positive and negative predictive values of FN for differential diagnosis of early-stage BC from control were obviously different because the validation set had only 57 patients with early-stage BC, compared with 119 in the test set. The sensitivity, specificity, and positive likelihood ratio of FN also differed between sets (Table 2). These findings can be explained by the difference in terms of the sample size and the proportion of patients with early-stage BC between the validation and the test sets (Table 2 and 3). Despite these differences, the diagnostic capabilities of FN were generally similar in the two sets. Moreover, this result indicated the levels of FN were irrelevant to subtype of BC for diagnosis in patients with early BC and prognosis in BC patients after surgical resection.

Many studies on function and expression of FN in cancer cells have been reported. This protein has been found to be expressed in BC [40, 41], and other cancers [37, 42, 43]. Moreover, it has been reported that FN could induce progression of various cancer cells [36, 37] and is strongly expressed in breast carcinoma, and its distribution is different from that of normal breast parenchyma [38, 39]. Although measurement of FN levels in other cancer groups is necessary, we have limitation in this study to collect patients' plasma with other cancers in hospital. In subsequent study, we will confirm FN level in other cancer groups.

Currently, BC has been diagnosed and prognosed by one or more methods such as mammograms, breast ultrasound, magnetic resonance imaging, or biopsy. However, these methods are often misleading and can be involved in expensive and painful methods. The test using FN in blood might improve up for these weak points in the current diagnosis and prognosis methods. Although the blood test developed in this study might well perform in a diagnostic setting with imaging data, there is a limitation for screening setting in current study because there is no evidence which the level of FN in plasma is increased in patients with the early stage of breast cancer only. It is required for further study to apply this method to screening setting. We are planning to collect large number of blood samples from women who visit hospital and perform the blood test to evaluate the level of FN in blood. It is expected that more biomarkers to detect specifically BC could be required and thus the further discovery of molecular biomarkers might be necessary to generate a panel of biomarkers for usage in screening setting.

Meanwhile, our study is cross-sectional and retrospective in nature and, therefore, we plan to do a prospective study to assess whether use of FN can be validated in patients with BC. The striking decrease in FN concentrations in plasma after surgery suggests that this protein could be useful prognostic biomarkers to assess the therapeutic response of BC patients. To further explore this potential role, we plan to undertake long-term follow-up of the BC patients who underwent surgery with BC. Furthermore, this method should be investigated for the application to various clinical situations, including evaluation for the response of chemotherapy, the early detection of recurrence after surgical resection and chemotherapy, and the monitoring of high risk groups for breast cancer. It will be a great method if this simple blood test could reduce the frequency and the cost for current imaging tests.

To our knowledge, this is the first study to report the diagnostic relevance of FN as plasma EV protein markers for BC in a test set and an independent validation set. The amount of FN on EVs in a small amount of plasma (1 μl) could be determined without EV purification in Method 1. This assay is simple, reproducible, quantitative, and non-invasive, and provides a highly reliable and sensitive indication as to the presence of BC. Overall, studies on a disease-specific protein on the surface of EVs, which are found in plasma from patients in the early stages breast cancer, provides the potential to facilitate the development of excellent biomarkers for various cancers and can be viewed as an emerging field in cancer biology. In addition, FN on EVs might offer a new therapeutic target for the treatment of BC.

## MATERIALS AND METHODS

### Study design

An overview of the study design used to identify biomarkers of BC is illustrated in Figure 2. We used plasma samples from 415 patients and 70 healthy volunteers in this study. We recruited plasma from patients and healthy controls, from Kyungpook National University Hospital (KNUH), Daegu, Korea and Chonnam National University Hwasun Hospital, Hwasun, Korea. The demographics, histological cell type, and stage of BC of the patients studied in test and validation sets are provided in Table 1.

FN were selected as a potential biomarker for BC. Using plasma from a 150 patients with BC and 30 healthy control (test set), we used to test differential expression of diagnostic marker candidates using two different types of ELISA method. The test set contained 20 patients with benign breast tumor, 40 patients with BC after surgical resection of their tumor, and 30 patients with non-cancerous diseases (thyroiditis, gastritis, hepatitis B, and rheumatoid arthritis). To validate biomarker candidates for BC, levels of FN were measured in 90 patients with BC, 40 healthy control, 35 patients with benign breast tumor, and 50 patients with non-cancerous diseases using two types of ELISAs (validation set). We used tumor, node and metastasis (TNM) Classification from the 7th edition of the American Joint Committee on Cancer (AJCC) to define early-stage BC (0, I and II) [44].

All individuals provided informed consent for blood donation according to a protocol approved by the institutional review board of KNUH.

### Proteomic analysis

EVs from two BC cell lines resuspended in 100 mM triethylammonium bicarbonate (TEABC, pH 8.0) were reduced with 10 mM dithiothreitol (DTT) at 60°C for 20 min, alkylated with 55 mM iodoacetamide (IAA) at room temperature for 30 min, and subjected to digestion for trypsin treatment [34]. The digested peptides were desalted using an hydrophilic lipophilic balanced (HLB) cartridge (Waters Oasis). The peptides were analyzed by nano-ultra performance liquid chromatography (UPLC) (Waters) and mass spectrometry using quadrupole-time-of-flight (Q-Tof) Premier (Waters). Data processing, searching, and analysis were performed using Mascot server 2.2 (Matrix Science).

### ELISA

For quantification of FN proteins on EVs in plasma using ELISA Method 1, 96-well plates were coated with polyclonal anti-CD63 (ab68418; Abcam) antibody at 100 ng/well in sodium phosphate buffer. The plates were blocked for 1 h at 37°C with of phosphate-buffered saline (PBS) containing 1% bovine serum albumin (BSA), and washed three times with PBS containing 0.05% Tween 20 (PBS-T). Plasma (10 μL) was diluted with blocking buffer (90 μL). The diluted plasma (10 μL) was added to the plates in triplicate and incubated for 1 h at 37°C. Following washes with PBS-T, the plates were reacted with monoclonal anti-FN (ab25583; Abcam) antibody, pre-incubated with peroxidase conjugated anti-mouse IgG antibody for 30 min, and developed with 3,3′,5,5′-tetramethylbenzidine containing hydrogen peroxide. The reaction was stopped with 1 M phosphoric acid and optical density values were measured at 450 nm on an iMark plate reader (BioRad).

For quantification of FN, or CD63 proteins in plasma using ELISA Method 2, the same steps were performed as with Method 1 except for coating with polyclonal anti-FN (ab23750; Abcam), or anti-CD63 (ab68418; Abcam) antibodies to capture each protein. FN (4305-FN-200; R&D Systems), or CD63 proteins (H00000967-G01; Abnova) were used to generate standard curves. The levels of FN or CD63 were measured using a monoclonal anti-FN (ab25583; Abcam) or anti-CD63 (ab8219; Abcam) antibody. Each data point is the average of triplicate measurements.

### Statistical analyses

Descriptive statistics summarized clinical factors *χ*^2^ and *t* tests were used to compare the test and validation groups. For FN and CD63 levels, relationships were analyzed using the unpaired two-tailed *t*-test with Welch's correction to assess differences between two groups. Assessment of the correlation between tumor size and levels of FN were performed using a Spearman correlation. The diagnostic potential of FN was determined by calculating the receiver operating characteristic (ROC) curve that was plotted to evaluate the sensitivity and specificity of the measurements in predicting BC. For evaluation of a significant change of FN in the presence of three types of receptors related to BC, Kruskal-Wallis one-way analysis was used. All *p* values of less than .05 are considered to indicate statistical significance. All analyses were calculated using MedCalc (MedCalc Software) and Prism (GraphPad Software, Inc.).

## SUPPLEMENTARY FIGURES AND TABLES









## References

[1] Acharyya S, Oskarsson T, Vanharanta S, Malladi S, Kim J, Morris PG, Manova-Todorova K, Leversha M, Hogg N, Seshan VE, Norton L, Brogi E, Massague J (2012). A CXCL1 paracrine network links cancer chemoresistance and metastasis. Cell.

[2] Maxmen A (2012). The hard facts. Nature.

[3] Polyak K (2006). Pregnancy and breast cancer: the other side of the coin. Cancer cell.

[4] Etzioni R, Urban N, Ramsey S, McIntosh M, Schwartz S, Reid B, Radich J, Anderson G, Hartwell L (2003). The case for early detection. Nature reviews Cancer.

[5] Weigelt B, Peterse JL, van 't Veer LJ (2005). Breast cancer metastasis: markers and models. Nature reviews Cancer.

[6] Hanahan D, Weinberg RA (2000). The hallmarks of cancer. Cell.

[7] Friedl P, Wolf K (2003). Tumour-cell invasion and migration: diversity and escape mechanisms. Nature reviews Cancer.

[8] Ginsberg MH, Partridge A, Shattil SJ (2005). Integrin regulation. Current opinion in cell biology.

[9] Juliano RL, Reddig P, Alahari S, Edin M, Howe A, Aplin A (2004). Integrin regulation of cell signalling and motility. Biochemical Society transactions.

[10] Cukierman E, Pankov R, Yamada KM (2002). Cell interactions with three-dimensional matrices. Current opinion in cell biology.

[11] Sieg DJ, Hauck CR, Ilic D, Klingbeil CK, Schaefer E, Damsky CH, Schlaepfer DD (2000). FAK integrates growth-factor and integrin signals to promote cell migration. Nature cell biology.

[12] Yeatman TJ (2004). A renaissance for SRC. Nature reviews Cancer.

[13] Irie HY, Pearline RV, Grueneberg D, Hsia M, Ravichandran P, Kothari N, Natesan S, Brugge JS (2005). Distinct roles of Akt1 and Akt2 in regulating cell migration and epithelial-mesenchymal transition. The Journal of cell biology.

[14] Han S, Ritzenthaler JD, Sitaraman SV, Roman J (2006). Fibronectin increases matrix metalloproteinase 9 expression through activation of c-Fos via extracellular-regulated kinase and phosphatidylinositol 3-kinase pathways in human lung carcinoma cells. The Journal of biological chemistry.

[15] Livant DL, Brabec RK, Kurachi K, Allen DL, Wu Y, Haaseth R, Andrews P, Ethier SP, Markwart S (2000). The PHSRN sequence induces extracellular matrix invasion and accelerates wound healing in obese diabetic mice. The Journal of clinical investigation.

[16] Gaggioli C, Robert G, Bertolotto C, Bailet O, Abbe P, Spadafora A, Bahadoran P, Ortonne JP, Baron V, Ballotti R, Tartare-Deckert S (2007). Tumor-derived fibronectin is involved in melanoma cell invasion and regulated by V600E B-Raf signaling pathway. The Journal of investigative dermatology.

[17] Muralidharan-Chari V, Clancy JW, Sedgwick A, D'Souza-Schorey C (2010). Microvesicles: mediators of extracellular communication during cancer progression. Journal of cell science.

[18] Raposo G, Stoorvogel W (2013). Extracellular vesicles: exosomes, microvesicles, and friends. The Journal of cell biology.

[19] Peinado H, Aleckovic M, Lavotshkin S, Matei I, Costa-Silva B, Moreno-Bueno G, Hergueta-Redondo M, Williams C, Garcia-Santos G, Ghajar C, Nitadori-Hoshino A, Hoffman C, Badal K (2012). Melanoma exosomes educate bone marrow progenitor cells toward a pro-metastatic phenotype through MET. Nature medicine.

[20] Luga V, Zhang L, Viloria-Petit AM, Ogunjimi AA, Inanlou MR, Chiu E, Buchanan M, Hosein AN, Basik M, Wrana JL (2012). Exosomes mediate stromal mobilization of autocrine Wnt-PCP signaling in breast cancer cell migration. Cell.

[21] Antonyak MA, Li B, Boroughs LK, Johnson JL, Druso JE, Bryant KL, Holowka DA, Cerione RA (2011). Cancer cell-derived microvesicles induce transformation by transferring tissue transglutaminase and fibronectin to recipient cells. Proceedings of the National Academy of Sciences of the United States of America.

[22] Taylor DD, Gercel-Taylor C (2011). Exosomes/microvesicles: mediators of cancer-associated immunosuppressive microenvironments. Seminars in immunopathology.

[23] Hosseini-Beheshti E, Pham S, Adomat H, Li N, Tomlinson Guns ES (2012). Exosomes as biomarker enriched microvesicles: characterization of exosomal proteins derived from a panel of prostate cell lines with distinct AR phenotypes. Mol Cell Proteomics.

[24] D'Souza-Schorey C, Clancy JW (2012). Tumor-derived microvesicles: shedding light on novel microenvironment modulators and prospective cancer biomarkers. Genes & development.

[25] Moon PG, You S, Lee JE, Hwang D, Baek MC (2011). Urinary exosomes and proteomics. Mass spectrometry reviews.

[26] Cho YE, Singh TS, Lee HC, Moon PG, Lee JE, Lee MH, Choi EC, Chen YJ, Kim SH, Baek MC (2012). In-depth identification of pathways related to cisplatin-induced hepatotoxicity through an integrative method based on an informatics-assisted label-free protein quantitation and microarray gene expression approach. Mol Cell Proteomics. MCP.

[27] Zhang Y, Fonslow BR, Shan B, Baek MC, Yates JR (2013). Protein analysis by shotgun/bottom-up proteomics. Chemical reviews.

[28] Taylor DD, Gercel-Taylor C (2008). MicroRNA signatures of tumor-derived exosomes as diagnostic biomarkers of ovarian cancer. Gynecologic oncology.

[29] Schipper HM, Maes OC, Chertkow HM, Wang E (2007). MicroRNA expression in Alzheimer blood mononuclear cells. Gene Regul Syst Bio.

[30] Vaz C, Ahmad HM, Sharma P, Gupta R, Kumar L, Kulshreshtha R, Bhattacharya A (2010). Analysis of microRNA transcriptome by deep sequencing of small RNA libraries of peripheral blood. BMC Genomics.

[31] Sheinerman KS, Tsivinsky VG, Crawford F, Mullan MJ, Abdullah L, Umansky SR (2012). Plasma microRNA biomarkers for detection of mild cognitive impairment. Aging (Albany NY).

[32] Tsujiura M, Ichikawa D, Komatsu S, Shiozaki A, Takeshita H, Kosuga T, Konishi H, Morimura R, Deguchi K, Fujiwara H, Okamoto K, Otsuji E (2010). Circulating microRNAs in plasma of patients with gastric cancers. Br J Cancer.

[33] Melo SA, Luecke LB, Kahlert C, Fernandez AF, Gammon ST, Kaye J, LeBleu VS, Mittendorf EA, Weitz J, Rahbari N, Reissfelder C, Pilarsky C, Fraga MF (2015). Glypican-1 identifies cancer exosomes and detects early pancreatic cancer. Nature.

[34] Moon PG, Lee JE, You S, Kim TK, Cho JH, Kim IS, Kwon TH, Kim CD, Park SH, Hwang D, Kim YL, Baek MC (2011). Proteomic analysis of urinary exosomes from patients of early IgA nephropathy and thin basement membrane nephropathy. Proteomics.

[35] Chen CL, Lai YF, Tang P, Chien KY, Yu JS, Tsai CH, Chen HW, Wu CC, Chung T, Hsu CW, Chen CD, Chang YS, Chang PL (2012). Comparative and targeted proteomic analyses of urinary microparticles from bladder cancer and hernia patients. J Proteome Res.

[36] Gorczyca W, Holm R, Nesland JM (1993). Laminin production and fibronectin immunoreactivity in breast carcinomas. Anticancer research.

[37] Han S, Khuri FR, Roman J (2006). Fibronectin stimulates non-small cell lung carcinoma cell growth through activation of Akt/mammalian target of rapamycin/S6 kinase and inactivation of LKB1/AMP-activated protein kinase signal pathways. Cancer research.

[38] Berry SD, Howard RD, Akers RM (2003). Mammary localization and abundance of laminin, fibronectin, and collagen IV proteins in prepubertal heifers. J Dairy Sci.

[39] Li W, Liu Z, Zhao C, Zhai L (2015). Binding of MMP-9-degraded fibronectin to beta6 integrin promotes invasion via the FAK-Src-related Erk1/2 and PI3K/Akt/Smad-1/5/8 pathways in breast cancer. Oncol Rep.

[40] Christensen L (1992). The distribution of fibronectin, laminin and tetranectin in human breast cancer with special attention to the extracellular matrix. APMIS Suppl.

[41] Fernandez-Garcia B, Eiro N, Marin L, Gonzalez-Reyes S, Gonzalez LO, Lamelas ML, Vizoso FJ (2014). Expression and prognostic significance of fibronectin and matrix metalloproteases in breast cancer metastasis. Histopathology.

[42] Jia D, Entersz I, Butler C, Foty RA (2012). Fibronectin matrix-mediated cohesion suppresses invasion of prostate cancer cells. BMC Cancer.

[43] Kenny HA, Chiang CY, White EA, Schryver EM, Habis M, Romero IL, Ladanyi A, Penicka CV, George J, Matlin K, Montag A, Wroblewski K, Yamada SD (2014). Mesothelial cells promote early ovarian cancer metastasis through fibronectin secretion. The Journal of clinical investigation.

[44] Gnant M, Mlineritsch B, Stoeger H, Luschin-Ebengreuth G, Heck D, Menzel C, Jakesz R, Seifert M, Hubalek M, Pristauz G, Bauernhofer T, Eidtmann H, Eiermann W (2011). Adjuvant endocrine therapy plus zoledronic acid in premenopausal women with early-stage breast cancer: 62-month follow-up from the ABCSG-12 randomised trial. The Lancet Oncology.

