# Supplementary material for: Fibronectin on circulating extracellular vesicles as a liquid biopsy to detect breast cancer
Source: Oncotarget. 2016 May 23;7(26):40189–99. doi: 10.18632/oncotarget.9561 (PMC5130002; doi:10.18632/oncotarget.9561)
Supplement: Supplementary file 1 [file oncotarget-07-40189-s001.pdf]

Fibronectin on circulating extracellular vesicles as a liquid biopsy to detect breast cancer

SUPPLEMENTARY FIGURES AND TABLES

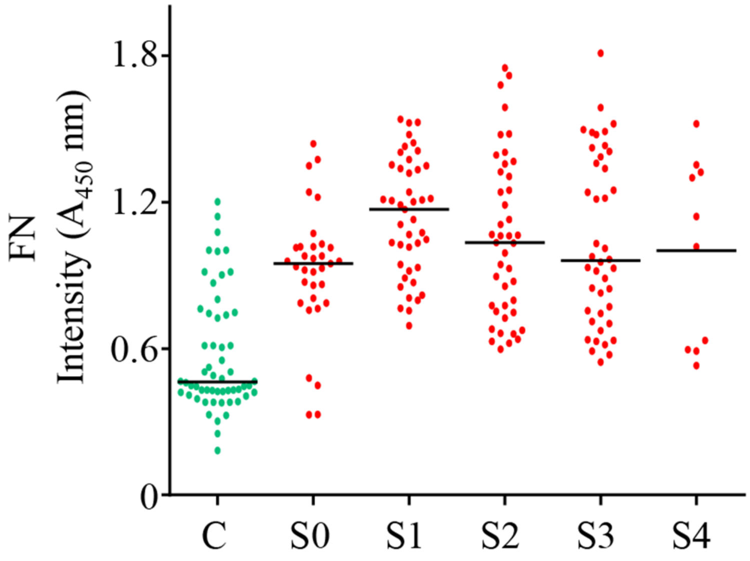

Supplementary Figure S1: Fibronectin levels in plasma from health control and patients with each stage breast cancer in the test set using enzyme-linked immunosorbent assays. Healthy controls (HC), *n* = 30; stage 0 (S0), *n* = 33; stage I (S1), *n* = 43; stage II (S2), *n* = 43; stage III (S3), *n* = 21; stage IV (S4), *n* = 10. The black horizontal lines are means, and error bars are SEs.

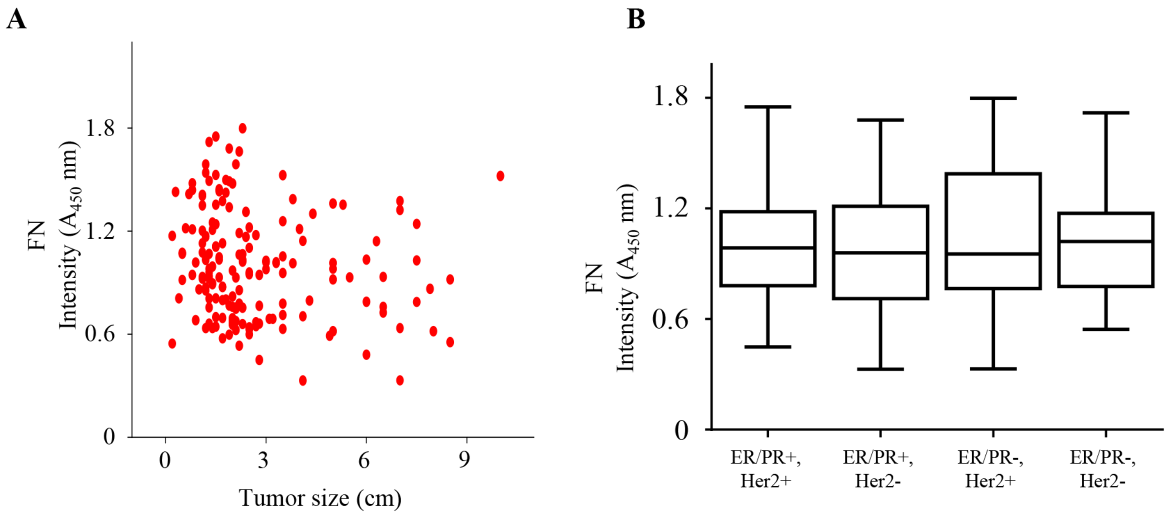

Supplementary Figure S2: A. The correlation between fibronectin (FN) levels and tumor size. There was no correlation between the levels of FN and tumor size. **B.** FN levels and four types of receptor status associated with breast cancer in test set. ER/PR+Her2+ (ER+/PR+Her2+, ER+/PR-Her2+, or ER-/PR+Her2+), n = 18; ER/PR+Her2- (ER+/PR+Her2-, ER-/PR+Her2-, or ER+/PR-Her2-), n = 111; ER/PR-Her2+ (ER-/PR-Her2+) n = 16; ER/PR-Her2- (ER-/PR-Her2-), n = 24. The black lines indicate the medians.

Supplementary Table S1: List of proteins identified EVs derived from the breast cancer cell line MDA-MB-231. For each protein, they show IPI accession number, protein description, Mascot score, protein mass, and identified peptides.

See Supplementary File 1

Supplementary Table S2: List of proteins identified EVs derived from the breast cancer cell line MCF-7. For each protein, they show IPI accession number, protein description, Mascot score, protein mass, and identified peptides.

See Supplementary File 2

Supplementary Table S3: List of proteins identified both EVs from MDA-MB-231 and MCF-7. For each protein, they show IPI accession number and protein description.

See Supplementary File 3
