# Supplementary material for: Fibronectin on circulating extracellular vesicles as a liquid biopsy to detect breast cancer
Source: Oncotarget. 2016 May 23;7(26):40189–99. doi: 10.18632/oncotarget.9561 (PMC5130002; doi:10.18632/oncotarget.9561)
Supplement: Supplementary file 2 [file oncotarget-07-40189-s002.docx]

**Table 1.** Characteristics of Study Population

| Characteristic | Test | | | | Validation | | | | Total | | *P^*^* |
| --- | --- | --- | --- | --- | --- | --- | --- | --- | --- | --- | --- |
|  | No. | % | Mean | SD | No. | % | Mean | SD | No. | % |  |
| Number of Study Population | 270 | 56.9 |  |  | 215 | 43.1 |  |  | 485 |  |  |
| Age, years |  |  | 51.2 | 11.4 |  |  | 51.9 | 12.3 |  |  | .8090^**^ |
| Breast cancer | 150 | 62.8 |  |  | 90 | 37.2 |  |  | 240 |  |  |
| Histological grade^†^ |  |  |  |  |  |  |  |  |  |  | .0148^#^ |
| 1  2  3 | 24  89  37 | 17.9  56.8  25.4 |  |  | 16  36  38 | 19.0  40.0  41.0 |  |  | 40  125  75 | 18.2  50.6  31.2 |  |
| Stage^‡^ |  |  |  |  |  |  |  |  |  |  | <.0001^#^ |
| 0  I  II  III  IV | 33  43  43  21  10 | 22.0  28.7  28.7  14.0  6.6 |  |  | 4  15  38  33 | 5.0  18.0  42.0  35.0 |  |  | 37  58  81  54  10 | 15.4  24.2  33.7  22.5  4.2 |  |
| Estrogen receptor (ER) ^¶^ |  |  |  |  |  |  |  |  |  |  | .2045^#^ |
| Negative  Positive | 33  117 | 24.9  75.1 |  |  | 27  63 | 32.0  68.0 |  |  | 60  180 | 27.5  72.5 |  |
| Progesterone receptor (PgR) ^¶^ |  |  |  |  |  |  |  |  |  |  | .7049^#^ |
| Negative  Positive | 47  103 | 33.7  66.3 |  |  | 31  59 | 36.0  64.0 |  |  | 78  162 | 34.6  65.4 |  |
| HER2^¶^ |  |  |  |  |  |  |  |  |  |  | .4544^#^ |
| Positive^§^  Negative | 24  126 | 20.1  79.9 |  |  | 21  69 | 24.0  76.0 |  |  | 45  195 | 21.6  78.4 |  |
| Healthy control | 30 | 43.2 |  |  | 40 | 56.8 |  |  | 70 |  |  |
| Non-cancerous diseases | 30 |  |  |  | 50 |  |  |  | 80 |  | .4635^#^ |
| Thyroiditis  Gastritis  Hepatitis B  Rheumatoid arthritis | 5  5  4  16 | 20  17.5  15  47.5 |  |  | 7  5  5  33 | 13.8  12.1  10.3  63.8 |  |  | 12  12  9  49 | 16.3  14.3  12.2  57.1 |  |
| After surgery | 40 |  |  |  |  |  |  |  | 40 |  |  |
| Benign breast tumor | 20 | 40.6 |  |  | 35 | 59.4 |  |  | 55 |  |  |

^*^ *P* value comparing test and validation groups. ^**^ *t*-test. ^#^ *χ*^2^ test.

^†^modified Scarff-Bloom-Richardson grading system. ^‡^7th edition of AJCC staging system. ^¶^according to immunohistochemical (IHC) staining of ER, PgR, and HER2. ^§^IHC (3+) or FISH (+).
