# Supplementary material for: Fibronectin on circulating extracellular vesicles as a liquid biopsy to detect breast cancer
Source: Oncotarget. 2016 May 23;7(26):40189–99. doi: 10.18632/oncotarget.9561 (PMC5130002; doi:10.18632/oncotarget.9561)
Supplement: Supplementary file 3 [file oncotarget-07-40189-s003.docx]

**Table 2.** Results for measurement of plasma fibronectin using enzyme-linked immunosorbent assays (ELISA) in the diagnosis of breast cancer in test set

|  | AUC (95%CI) | Sensitivity (%) | Specificity (%) | LR + | NR - |
| --- | --- | --- | --- | --- | --- |
| Method 1 | | | | | |
| BC *vs* HC+bB+NC* | 0.810 (0.758-0.862) | 65.1% | 83.2% | 3.88 | 0.42 |
| BC *vs* bB+NC | 0.746 (0.680-0.811) | 63.4% | 78.8% | 2.99 | 0.46 |
| Early-BC *vs* HC+bB+NC | 0.815 (0.761-0.869) | 64.4% | 84.2% | 4.08 | 0.42 |
| Early- BC *vs* bB+NC | 0.754 (0.685-0.822) | 65.9% | 77.3% | 2.90 | 0.44 |
| Method 2 | | | | | |
| BC *vs* HC+bB+NC | 0.773(0.721-0.834) | 69.2% | 73.3% | 2.59 | 0.42 |
| BC *vs* bB+NC | 0.710 (0.641-0.779) | 60.4% | 75.8% | 2.49 | 0.52 |
| Early-BC *vs* HC+bB+NC | 0.779 (0.719-0.838) | 72.7% | 71.3% | 2.53 | 0.38 |
| Early- BC *vs* bB+NC | 0.713 (0.640-0.787) | 59.9% | 75.8% | 2.47 | 0.53 |

*BC, breast cancer; HC, Healthy controls; bB, benign breast tumors; NC, non-cancerous diseases; Early- BC, early stage breast cancer
