# Supplementary material for: Fibronectin on circulating extracellular vesicles as a liquid biopsy to detect breast cancer
Source: Oncotarget. 2016 May 23;7(26):40189–99. doi: 10.18632/oncotarget.9561 (PMC5130002; doi:10.18632/oncotarget.9561)
Supplement: Supplementary file 4 [file oncotarget-07-40189-s004.docx]

**Table 3.** Results for measurement of plasma fibronectin using enzyme-linked immunosorbent assays (ELISA) in the diagnosis of breast cancer in validation set

|  | AUC (95%CI) | Sensitivity (%) | Specificity (%) | LR + | NR - |
| --- | --- | --- | --- | --- | --- |
| Method 1 | | | | | |
| BC *vs* HC+bB+NC | 0.748 (0.683-0.812) | 68.9% | 72.0% | 2.43 | 0.44 |
| BC *vs* bB+NC | 0.736 (0.666-0.806) | 66.1% | 74.2% | 2.56 | 0.46 |
| Early-BC *vs* HC+bB+NC | 0.737 (0.657-0.812) | 67.7% | 72.0% | 2.42 | 0.45 |
| Early- BC *vs* bB+NC | 0.722 (0.637-0.807) | 67.7% | 74.3% | 2.63 | 0.43 |
| Method 2 | | | | | |
| BC *vs* HC+bB+NC | 0.684 (0.614-0.753) | 54.4% | 75.2% | 2.19 | 0.61 |
| BC *vs* bB+NC | 0.665 (0.589-0.741) | 56.0% | 75.7% | 2.30 | 0.58 |
| Early-BC *vs* HC+bB+NC | 0.672 (0.591-0.753) | 49.2% | 75.2% | 1.98 | 0.68 |
| Early- BC *vs* bB+NC | 0.654 (0.566-0.743) | 49.2% | 76.7% | 2.11 | 0.66 |

*BC, breast cancer; HC, Healthy controls; bB, benign breast tumors; NC, non-cancerous diseases; Early- BC, early stage breast cancer
